# *dfrA thyA* Double Deletion in *para*-Aminosalicylic Acid-Resistant Mycobacterium tuberculosis Beijing Strains

**DOI:** 10.1128/AAC.00253-16

**Published:** 2016-05-23

**Authors:** Danesh Moradigaravand, Louis Grandjean, Elena Martinez, Hao Li, Jun Zheng, Jorge Coronel, David Moore, M. Estée Török, Vitali Sintchenko, Hairong Huang, Babak Javid, Julian Parkhill, Sharon J. Peacock, Claudio U. Köser

**Affiliations:** a Wellcome Trust Sanger Institute, Hinxton, United Kingdom; b Wellcome Centre for Clinical Tropical Medicine, Imperial College London, St. Mary's Campus, London, United Kingdom; c London School of Hygiene and Tropical Medicine, London, United Kingdom; d Laboratorio de Investigación en Enfermedades Infecciosas, Universidad Peruana Cayetano Heredia, Lima, Peru; e Centenary Institute and Marie Bashir Institute for Infectious Diseases and Biosecurity, The University of Sydney, Sydney, Australia; f NSW Mycobacterium Reference Laboratory, Centre for Infectious Diseases and Microbiology Laboratory Services, Institute of Clinical Pathology and Medical Research-Pathology West, Sydney, Australia; g Centre for Infectious Diseases and Microbiology-Public Health, Westmead Hospital, Western Sydney Local Health District, Sydney, Australia; h Collaborative Innovation Centre for the Diagnosis and Treatment of Infectious Diseases, School of Medicine, Tsinghua University, Beijing, China; i Faculty of Health Sciences, University of Macau, Macau SAR, China; j Department of Medicine, University of Cambridge, Cambridge, United Kingdom; k Department of Infectious Diseases, Cambridge University Hospitals NHS Foundation Trust, Cambridge, United Kingdom; l Clinical Microbiology and Public Health Laboratory, Public Health England, Cambridge, United Kingdom; m National Clinical Laboratory on Tuberculosis, Beijing Key Laboratory on Drug-Resistant Tuberculosis Research, Beijing Chest Hospital, Capital Medical University, Beijing Tuberculosis and Thoracic Tumor Institute, Beijing, China

## LETTER

**FIG 1:**
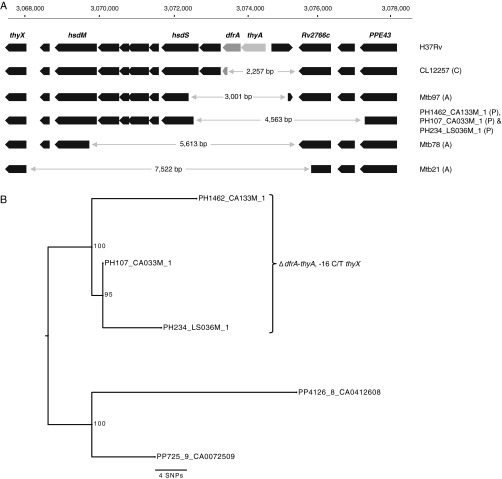
Analysis of *dfrA* and *thyA* deletion strains, all of which tested PAS resistant, with the exception of PH107_CA033M_1. (A) Diagram of deletions in seven clinical strains compared with the wild-type H37Rv laboratory strain. The scale at the top corresponds to the genome position in H37Rv. The letter in parentheses denotes the country of isolation (Australia [A], China [C], and Peru [P]). Mtb97 was reported previously (3). (B) Maximum likelihood tree based on whole-genome data of the three Peruvian deletion mutants, which also share a mutation upstream of *thyX* that is also present in Mtb97 and Mtb78 (Table S1), and two closely related wild-type strains, which were PAS susceptible.

*para*-Aminosalicylic acid (PAS) is a group 4 antituberculosis agent (1). It targets folate metabolism as shown in Fig. S1 in the supplemental material, which also summarizes the known mechanisms of resistance to this prodrug (2). Recently, we reported a multidrug-resistant (MDR) Mycobacterium tuberculosis Beijing strain harboring a deletion of both *dfrA* and *thyA* from Australia (Fig. 1A and Table S1) (3). Since then, we have found deletions affecting both genes in five further MDR Beijing strains (two isolated in Australia and three from Peru) and one extensively drug-resistant (XDR) Beijing strain from China. The Australian MDR strains were recovered from three patients with no apparent epidemiological links who were likely infected in their country of origin (Table S1). The three Peruvian isolates were closely related and consequently shared the same deletion, whereas the remaining strains were distantly related and had deletions that differed in size (Fig. 1A). Consequently, these five distinct deletions were acquired independently, which can be a signal for positive selection of resistance mechanisms. In line with this hypothesis, the strains from Australia and China were found to be PAS resistant when tested with the Bactec MGIT 960 system and on Löwenstein-Jensen medium, respectively (see Supplemental methods). Two out of the three Peruvian deletion mutants were also found to be PAS resistant on 7H10 medium at 8 μg/ml, whereas the two closely related ancestral wild-type strains were found to be susceptible (Fig. 1B). We were unable to retest the strains at 2 μg/ml, the critical concentration recommended by the Clinical and Laboratory Standards Institute and the World Health Organization, which would have clarified whether the result for the third deletion mutant as susceptible was an artifact (1, 4).

The observation that *dfrA* could be deleted was remarkable in light of our current understanding of folate metabolism in M. tuberculosis. Two studies suggested that *dfrA* is essential *in vitro* in the H37Rv laboratory strain (5, 6). More recently, it was shown that *dfrA* is conditionally essential and can be knocked out in H37Rv only if Rv2671 is overexpressed *in trans*, presumably due to its greatly reduced dihydrofolate reductase activity compared to that of DfrA (7, 8). Our *in silico* analysis of the seven *dfrA thyA* double deletion mutants did not reveal any known *Rv2671* mutations (Table S1), such as the G-to-A upstream mutation at position −12 that results in its overexpression and consequently confers PAS resistance (this mutation was incorrectly referred to as affecting position −11 in two of our prior studies [7, 9]). Assuming that no other pertinent differences that are specific to the Beijing genotype relative to H37Rv exist or that a yet-unknown acquired mutation elsewhere in the genome that resulted in the overexpression of *Rv2671* was present, we propose that this apparent contradiction can be reconciled if the essentiality of *dfrA* was dependent not only on the expression level of *Rv2671* but also on the presence of wild-type *thyA*. The fact that *thyA* was deleted in all seven *dfrA* mutants meant that only the second thymidylate synthase, encoded by the essential *thyX* gene, was active in these strains (Fig. S1). Contrary to ThyA, ThyX generates tetrahydrofolate rather than dihydrofolate upon catalysis and therefore does not require high dihydrofolate reductase activity to provide sufficient levels of tetrahydrofolate (2). This is in line with the fact that *dfrA* is not required in bacterial species that lack *thyA* (10). Consequently, *Rv2671* appeared to be sufficient to sustain growth, even without being overexpressed in these deletion mutants. It should therefore be possible to knock out *dfrA* in strains of M. tuberculosis with inactive *thyA*. Moreover, the adjacent locations of *thyA* and *dfrA* in the genome should make their simultaneous deletion possible (Fig. 1A).

Interestingly, all but one of the deletion mutants also convergently acquired mutations upstream of *thyX* compared to what was observed for the two closely related Peruvian control strains (Fig. 1B and Table S1) (11). In fact, the cluster of three Peruvian strains and two of the unrelated Australian strains shared the same C-to-T upstream mutation at position −16 that has previously been found to be associated with resistance to several drugs and experimentally shown to result in the overexpression of *thyX* (12). It is therefore plausible that these changes compensated for the reduced expression levels and enzymatic activity of ThyX compared to those of ThyA (11, 13). Based on our data, however, it was not possible to deduce whether the *thyX* mutations were acquired after the deletions of *thyA* and *dfrA* in each strain, as would be expected with compensatory mutations (11).

In summary, these data demonstrated that the folate metabolism and the genetic basis of PAS resistance are more complex than previously appreciated, which is relevant for the development of novel DfrA and ThyX inhibitors and potentially the use of trimethoprim-sulfamethoxazole to treat drug-resistant tuberculosis (Fig. S1) (14–25). Although deletions are often excluded from large-scale whole-genome studies, owing to the limited read lengths of next-generation sequencers and the fact that algorithms are optimized for single-nucleotide polymorphism (SNP) calling, this study highlighted that deletions can no longer be ignored (3, 26).

## Supplementary Material

Supplemental material
