# Supplementary material for: dfrA thyA Double Deletion in para-Aminosalicylic Acid-Resistant Mycobacterium tuberculosis Beijing Strains
Source: Antimicrob Agents Chemother. 2016 May 23;60(6):3864–7. doi: 10.1128/AAC.00253-16 (PMC4879365; doi:10.1128/AAC.00253-16)
Supplement: Supplemental material [file supp_60_6_3864__index.html]

dfrA thyA Double Deletion in para-Aminosalicylic Acid-Resistant Mycobacterium tuberculosis Beijing Strains — Supplemental material 

# *dfrA thyA* Double Deletion in *para*-Aminosalicylic Acid-Resistant Mycobacterium tuberculosis Beijing Strains

## Supplemental material

- Supplemental file 1 -

  Supplemental methods and Figure S1.

  PDF, 248K
- Supplemental file 2 -

  Table S1: overview of strains.

  XLSX, 11K
