# Supplementary material for: dfrA thyA Double Deletion in para-Aminosalicylic Acid-Resistant Mycobacterium tuberculosis Beijing Strains
Source: Antimicrob Agents Chemother. 2016 May 23;60(6):3864–7. doi: 10.1128/AAC.00253-16 (PMC4879365; doi:10.1128/AAC.00253-16)
Supplement: Supplemental material [file AAC.00253-16_zac006165228so1.pdf]

Supplementary Methods

Drug-susceptibility testing Australia
Stored *M. tuberculosis* isolates were recovered from -80°C and cultured on Middlebrook 7H10 agar. Drug susceptibility testing was performed using the BACTEC MGIT 960 system (Becton Dickinson) according to the manufacturer's instructions (1). The following critical concentrations were used to determine drug resistance: isoniazid 0.1 and 0.4 µg/mL, rifampicin 1 µg/mL, ethambutol 5 µg/mL, pyrazinamide 100 µg/mL, streptomycin 1 µg/mL, amikacin 1 µg/mL, capreomycin 2.5 µg/mL, ofloxacin 2 µg/mL, ethionamide 5 µg/mL, and PAS 4 µg/mL (using sodium 4-aminosalicylate dihydrate (2)). Testing was performed in the NSW Mycobacterium Reference Laboratory, Pathology West and Westmead Hospital, Sydney.

Drug-susceptibility testing China
Drug susceptibility testing was performed using the indirect proportion method on Löwenstein-Jensen medium (3). The following critical concentrations were used to determine drug resistance: isoniazid 0.2 µg/mL, rifampicin 40 µg/mL, ethambutol 2 µg/mL, streptomycin 4 µg/mL, kanamycin 30 µg/mL, ofloxacin 2 µg/mL, and PAS 1 µg/mL (using sodium 4-aminosalicylate dihydrate (2)). Three previously described PAS resistant strains (with a *thyA* H75N, *folC* R49W, and *ribD* G-12A mutation, respectively) served as positive controls and H37Rv as negative control (4).

Drug-susceptibility testing Peru
Drug susceptibility testing was undertaken using the indirect proportions method on 7H10 agar at the National Reference Laboratory of the Peruvian Institute of Health (2). The following critical concentrations were used to determine drug resistance: isoniazid 0.2 and 1 µg/mL, rifampicin 1.0 µg/mL, ethambutol 5 µg/mL, streptomycin 2 µg/mL, kanamycin 5 µg/mL, capreomycin 10 µg/mL, ciprofloxacin 2 µg/mL, ethionamide 5 µg/mL, cycloserine 30 µg/mL, and, PAS 8 µg/mL (using 4-amino-2-hydroxybenzoic acid) (5). Drug resistance to pyrazinamide was determined using the Wayne method (6).

Whole-genome sequencing and analysis of Chinese and Peruvian strains
We employed Illumina (San Diego, CA, USA) sequencing platforms at the Wellcome Trust Sanger Institute (Hinxton, UK) to obtain paired-end reads for the strains from China and Peru. Reads were mapped to the H37Rv reference genome published in Casali et al. using the aligner SMALT (7). Single-nucleotide polymorphisms (SNPs) and small insertions/deletions were detected using an in-house pipeline that combines bcftools and samtools (8). We used parameters in Harris et al. 2011 to call SNPs (9). The identified SNPs were then included in an SNP alignment, which was subsequently used to build a maximum likelihood phylogenetic tree using RAxML (10). Large deletions were detected using an in house tool at the Wellcome Trust Sanger Institute.

Ethical approval

Approval for whole-genome sequencing was granted by the UK National Research Ethics Service (12/EE/0439) and the Cambridge University Hospitals NHS Foundation Trust R&D Department (A092685). Ethical approval for the project in Peru was obtained from the Universidad Peruana Cayetano Heredia (IRB00001014) with approval number 57492 and institutional approval was obtained from the Peruvian Ministry of Health before the study was undertaken.

Figure S1. Overview of folate metabolism.

The pro-drug PAS acts as an alternative substrate for folate mabolism (starting with the dihydropteroate synthase FolP1 (Rv3608c) as shown on the left of the figure) and ultimately inhibits the dihydrofolate reductase that is encoded by *dfrA* (*Rv2763c*) and catalyses the conversion of dihydrofolate to tetrahydrofolate (11-13).

Three resistance mechanisms have been described to date. First, PAS efflux as a result of the over-expression of the efflux pump Tap (Rv1258c) results in increased minimum inhibition concentrations (14). Consequently, the over-expression of one of its positive regulators may result in PAS resistance (15). For example, the mutational over-expression of WhiB7 (Rv3197A) might lead to cross-resistance to kanamycin, streptomycin, as well as PAS (16, 17). The clinical relevance of this mechanism remains uncertain given that *tap* contains a frameshift mutation in most Beijing strains (16). Second, reduced bioactivation of PAS due to mutations in the dihydrofolate synthase FolC (Rv2447c) causes resistance (11, 12, 18). Third, mechanisms that mitigate the inhibition of DfrA confer PAS resistance. This can be achieved by (a) decreasing the catalytic demand on DfrA owing to loss-of-function mutations in the thymidylate synthase ThyA (Rv2764c), (b) the mutational over-expression of Rv2671, which is currently misannotated as the 5-amino-6-ribitylamino-2,4(1*H*,3*H*)-pyrimidinedione 5'-phosphate reductase RibD of the riboflavin biosynthetic pathway but actually is an alternative dihydrofolate reductase, or (c) the over-expression of *dfrA* itself, although this last mechanism has never been observed in clinical isolates (12, 13, 19-21). In fact, no DfrA mutations have been shown conclusively to confer PAS resistance (22). The three PAS resistance loci that have been found to be clinically relevant to date are marked with an asterisk and the potential compensatory gene *thyX* (*Rv2754c*) with a degree sign (4, 23). The two genes found to be deleted in this study are underlined (Fig. 1A & Table S1).

Trimethoprim also targets DfrA, as well as Rv2671, but is not active singly against *M. tuberculosis* (21). There are contradictory data about whether there are additive or synergistic effects between trimethoprim and sulfamethoxazole, the latter of which is active singly by inhibiting the dihydropteroate synthase FolP1 (24-26).

5,10-m-H_4PteGlu, 5,10-methylenetetrahydrofolate; DHPPP, 7,8-dihydropterin pyrophosphate; dTMP, deoxythymidine monophosphate; dUMP, deoxyuridine monophosphate; Glu, glutamate; H_2Pte, dihydropteroate; H_2PteGlu, dihydrofolate; H_4PteGlu, tetrahydrofolate; PABA, *para*-aminobenzoic acid; PAS, *para*-aminosalicylic acid; SMX, sulfamethoxazole; TMP, trimethoprim.

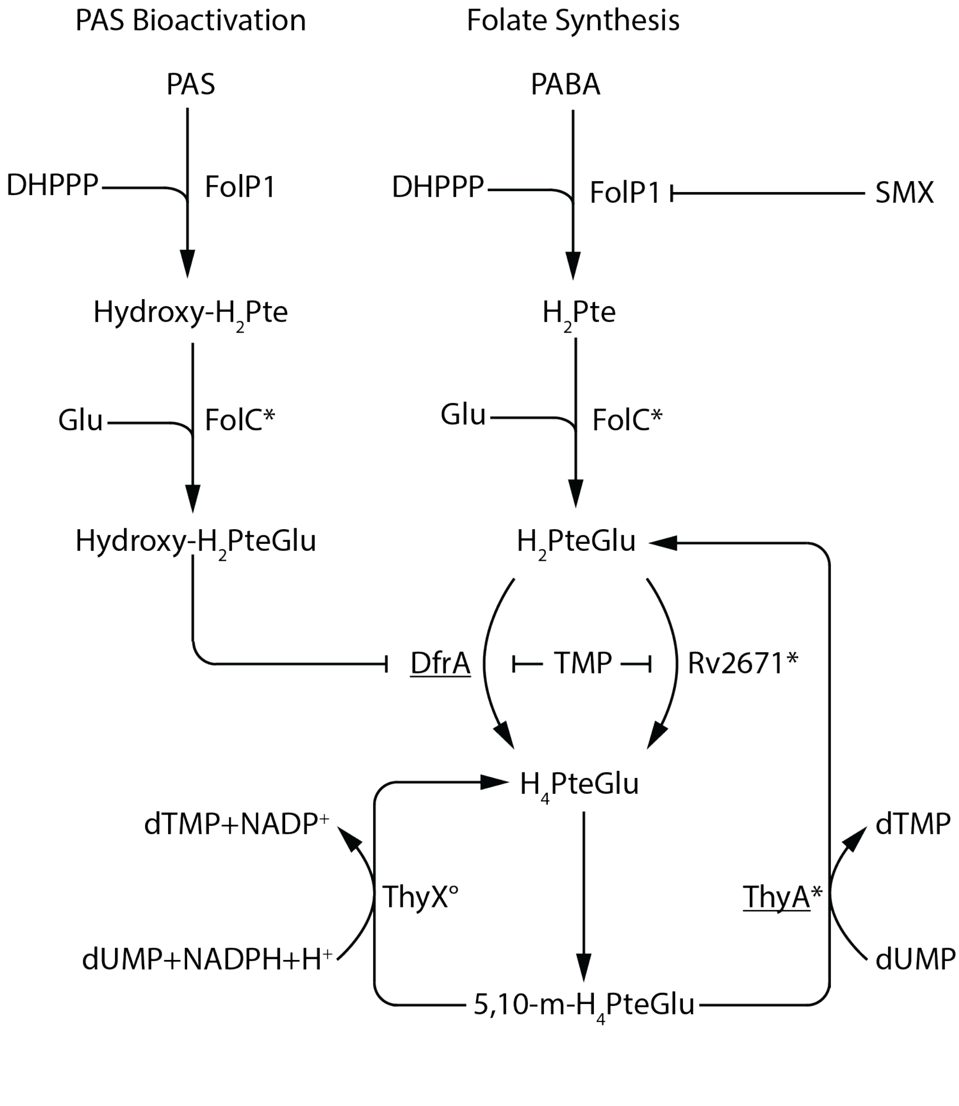

References

1. **Palomino JC, Traore H, Fissette K, Portaels F.** 1999. Evaluation of Mycobacteria Growth Indicator Tube (MGIT) for drug susceptibility testing of *Mycobacterium tuberculosis*. Int J Tuberc Lung Dis **3:**344-348.
2. **Canetti G, Froman S, Grosset J, Hauduroy P, Langerova M, Mahler HT, Meissner G, Mitchison DA, Sula L.** 1963. Mycobacteria: laboratory methods for testing drug sensitivity and resistance. Bull World Health Organ **29:**565-578.
3. **Organization WH.** Guidelines for drug susceptibility testing for second-line anti-tuberculosis drugs for DOTS-Plus. Geneva,Switzerland: WHO; 2001. WHO/CDS/TB/2001.288.
4. **Zhang X, Liu L, Zhang Y, Dai G, Huang H, Jin Q.** 2015. Genetic determinants involved in *p*-aminosalicylic acid resistance in clinical isolates from tuberculosis patients in northern China from 2006 to 2012. Antimicrob Agents Chemother **59:**1320-1324.
5. **Kent PT, Kubica GP.** 1985. Public health mycobacteriology. A guide for the level III laboratory.
6. **Wayne LG.** 1974. Simple pyrazinamidase and urease tests for routine identification of mycobacteria. Am Rev Respir Dis **109:**147-151.
7. **Casali N, Nikolayevskyy V, Balabanova Y, Ignatyeva O, Kontsevaya I, Harris SR, Bentley SD, Parkhill J, Nejentsev S, Hoffner SE, Horstmann RD, Brown T, Drobniewski F.** 2012. Microevolution of extensively drug-resistant tuberculosis in Russia. Genome Res **22:**735-745.
8. **Li H, Handsaker B, Wysoker A, Fennell T, Ruan J, Homer N, Marth G, Abecasis G, Durbin R.** 2009. The Sequence Alignment/Map format and SAMtools. Bioinformatics **25:**2078-2079.
9. **Harris SR, Feil EJ, Holden MT, Quail MA, Nickerson EK, Chantratita N, Gardete S, Tavares A, Day N, Lindsay JA, Edgeworth JD, de Lencastre H, Parkhill J, Peacock SJ, Bentley SD.** 2010. Evolution of MRSA during hospital transmission and intercontinental spread. Science **327:**469-474.
10. **Stamatakis A.** 2006. RAxML-VI-HPC: maximum likelihood-based phylogenetic analyses with thousands of taxa and mixed models. Bioinformatics **22:**2688-2690.
11. **Chakraborty S, Gruber T, Barry CE, 3rd, Boshoff HI, Rhee KY.** 2013. *Para*-aminosalicylic acid acts as an alternative substrate of folate metabolism in *Mycobacterium tuberculosis*. Science **339:**88-91.
12. **Zheng J, Rubin EJ, Bifani P, Mathys V, Lim V, Au M, Jang J, Nam J, Dick T, Walker JR, Pethe K, Camacho LR.** 2013. *Para*-aminosalicylic acid is a prodrug targeting dihydrofolate reductase in *Mycobacterium tuberculosis*. J Biol Chem **288:**23447-23456.
13. **Minato Y, Thiede JM, Kordus SL, McKlveen EJ, Turman BJ, Baughn AD.** 2015. *Mycobacterium tuberculosis* folate metabolism and the mechanistic basis for *para*-aminosalicylic acid susceptibility and resistance. Antimicrob Agents Chemother **59:**5097-5106.
14. **Ramón-García S, Mick V, Dainese E, Martin C, Thompson CJ, De Rossi E, Manganelli R, Aínsa JA.** 2012. Functional and genetic characterization of the tap efflux pump in *Mycobacterium bovis* BCG. Antimicrob Agents Chemother **56:**2074-2083.
15. **Rustad TR, Minch KJ, Ma S, Winkler JK, Hobbs S, Hickey M, Brabant W, Turkarslan S, Price ND, Baliga NS, Sherman DR.** 2014. Mapping and manipulating the *Mycobacterium tuberculosis* transcriptome using a transcription factor overexpression-derived regulatory network. Genome Biol **15:**502.
16. **Köser CU, Bryant JM, Parkhill J, Peacock SJ.** 2013. Consequences of *whiB7* (*Rv3197A*) mutations in Beijing genotype isolates of the *Mycobacterium tuberculosis* complex. Antimicrob Agents Chemother **57:**3461.
17. **Reeves AZ, Campbell PJ, Sultana R, Malik S, Murray M, Plikaytis BB, Shinnick TM, Posey JE.** 2013. Aminoglycoside cross-resistance in *Mycobacterium tuberculosis* due to mutations in the 5' untranslated region of *whiB7*. Antimicrob Agents Chemother **57:**1857-1865.
18. **Zhao F, Wang XD, Erber LN, Luo M, Guo AZ, Yang SS, Gu J, Turman BJ, Gao YR, Li DF, Cui ZQ, Zhang ZP, Bi LJ, Baughn AD, Zhang XE, Deng JY.** 2014. Binding pocket alterations in dihydrofolate synthase confer resistance to *para*-aminosalicylic acid in clinical isolates of *Mycobacterium tuberculosis*. Antimicrob Agents Chemother **58:**1479-1487.
19. **Rengarajan J, Sassetti C, Naroditskaya V, Sloutsky A, Bloom B, Rubin E.** 2004. The folate pathway is a target for resistance to the drug *para*-aminosalicylic acid (PAS) in mycobacteria. Mol Microbiol **53:**275-282.
20. **Fivian-Hughes AS, Houghton J, Davis EO.** 2012. *Mycobacterium tuberculosis* thymidylate synthase gene *thyX* is essential and potentially bifunctional, while *thyA* deletion confers resistance to *p*-aminosalicylic acid. Microbiology **158:**308-318.
21. **Cheng YS, Sacchettini JC.** 2016. Structural insights into *Mycobacterium tuberculosis* Rv2671 protein as a dihydrofolate reductase functional analogue contributing to *para*-aminosalicylic acid resistance. Biochemistry **55:**1107-1119.
22. **Mathys V, Wintjens R, Lefevre P, Bertout J, Singhal A, Kiass M, Kurepina N, Wang XM, Mathema B, Baulard A, Kreiswirth BN, Bifani P.** 2009. Molecular genetics of *para*-aminosalicylic acid resistance in clinical isolates and spontaneous mutants of *Mycobacterium tuberculosis*. Antimicrob Agents Chemother **53:**2100-2109.
23. **Merker M, Kohl TA, Roetzer A, Truebe L, Richter E, Rüsch-Gerdes S, Fattorini L, Oggioni MR, Cox H, Varaine F, Niemann S.** 2013. Whole genome sequencing reveals complex evolution patterns of multidrug-resistant *Mycobacterium tuberculosis* Beijing strains in patients. PLoS One **8:**e82551.
24. **Vilchèze C, Jacobs WR, Jr.** 2012. The combination of sulfamethoxazole, trimethoprim, and isoniazid or rifampin is bactericidal and prevents the emergence of drug resistance in *Mycobacterium tuberculosis*. Antimicrob Agents Chemother **56:**5142-5148.

25. **Huang TS, Kunin CM, Yan BS, Chen YS, Lee SS, Syu W, Jr.** 2012. Susceptibility of *Mycobacterium tuberculosis* to sulfamethoxazole, trimethoprim and their combination over a 12 year period in Taiwan. J Antimicrob Chemother **67:**633-637.

26. **Macingwana L, Baker B, Ngwane AH, Harper C, Cotton MF, Hesseling A, Diacon AH, van Helden P, Wiid I.** 2012. Sulfamethoxazole enhances the antimycobacterial activity of rifampicin. J Antimicrob Chemother **67:**2908-2911.
